# Percutaneous Nephrolithotomy in an Intrathoracic Kidney: A Rare Anatomical Variant With a Successful Outcome

**DOI:** 10.7759/cureus.100484

**Published:** 2025-12-31

**Authors:** Sarthak Sharma, Neeraj Agarwal, Karan Garg, Rajesh K Kumawat, Dharmendra K Jangid

**Affiliations:** 1 Urology and Renal Transplant Surgery, Sawai Man Singh (SMS) Medical College, Jaipur, IND

**Keywords:** congential diaphragmatic hernia, intrathoracic kidney, percutaneous nephrolithotomy, percutaneous nephrolithotomy (pcnl), renal ectopia, staghorn calculus

## Abstract

Thoracic kidney is the rarest form of renal ectopia and is usually detected incidentally. The coexistence of nephrolithiasis within an intrathoracic kidney associated with a congenital diaphragmatic hernia is extremely uncommon.

We report a 32-year-old woman with a left intrathoracic kidney containing a large staghorn calculus herniating through a congenital diaphragmatic defect. A percutaneous nephrostomy was placed under ultrasound and computed tomography (CT) guidance, and percutaneous nephrolithotomy (PCNL) was performed through the 9th intercostal space using a pneumatic lithotripter, achieving complete clearance. The patient had an uneventful recovery with no respiratory complications.

This rare case demonstrates that with meticulous imaging-based planning and multidisciplinary collaboration, minimally invasive management such as PCNL can be safely and effectively performed in intrathoracic kidneys with complex calculi.

## Introduction

A kidney present at any site other than the normal retroperitoneal renal fossa is called an ectopic kidney. Such kidneys may be positioned lower, higher, or even on the contralateral side. The incidence is approximately one in 12,000 clinical cases and one in 900 autopsies. An ectopic kidney is usually asymptomatic and may function normally despite its abnormal location. They are often incidentally detected during investigations performed for unrelated reasons [1].

An Intrathoracic kidney refers to the presence of renal tissue within the thoracic cavity. It presents the rarest form of renal ectopia and is usually asymptomatic, usually discovered incidentally. The incidence of intrathoracic kidney with the congenital diaphragmatic hernia is only 0.25% [2]. The occurrence of nephrolithiasis within an intrathoracic kidney, particularly when associated with a congenital diaphragmatic hernia, is exceedingly rare, with only a few cases described in the literature.

The major challenge in these cases lies in surgical planning, given the close proximity of the kidney to vital thoracic structures such as the heart, lungs, and major vessels. While the diaphragmatic defect itself typically does not necessitate surgical repair, the presence of symptomatic renal calculi requires definitive intervention.

We report a rare case of a large staghorn calculus in a left ectopic intrathoracic kidney associated with a congenital diaphragmatic hernia, successfully managed by percutaneous nephrolithotomy (PCNL).

## Case presentation

A 32-year-old woman presented with complaints of bilateral flank pain for four months and burning micturition for seven days. She had no known comorbidities. The patient had undergone right retrograde intrarenal surgery (RIRS) with bilateral double-J (DJ) stenting at another institution, followed by right stent removal one month later. She was referred to our centre for the management of a left intrathoracic kidney harbouring a large staghorn calculus.

**Figure 1:**
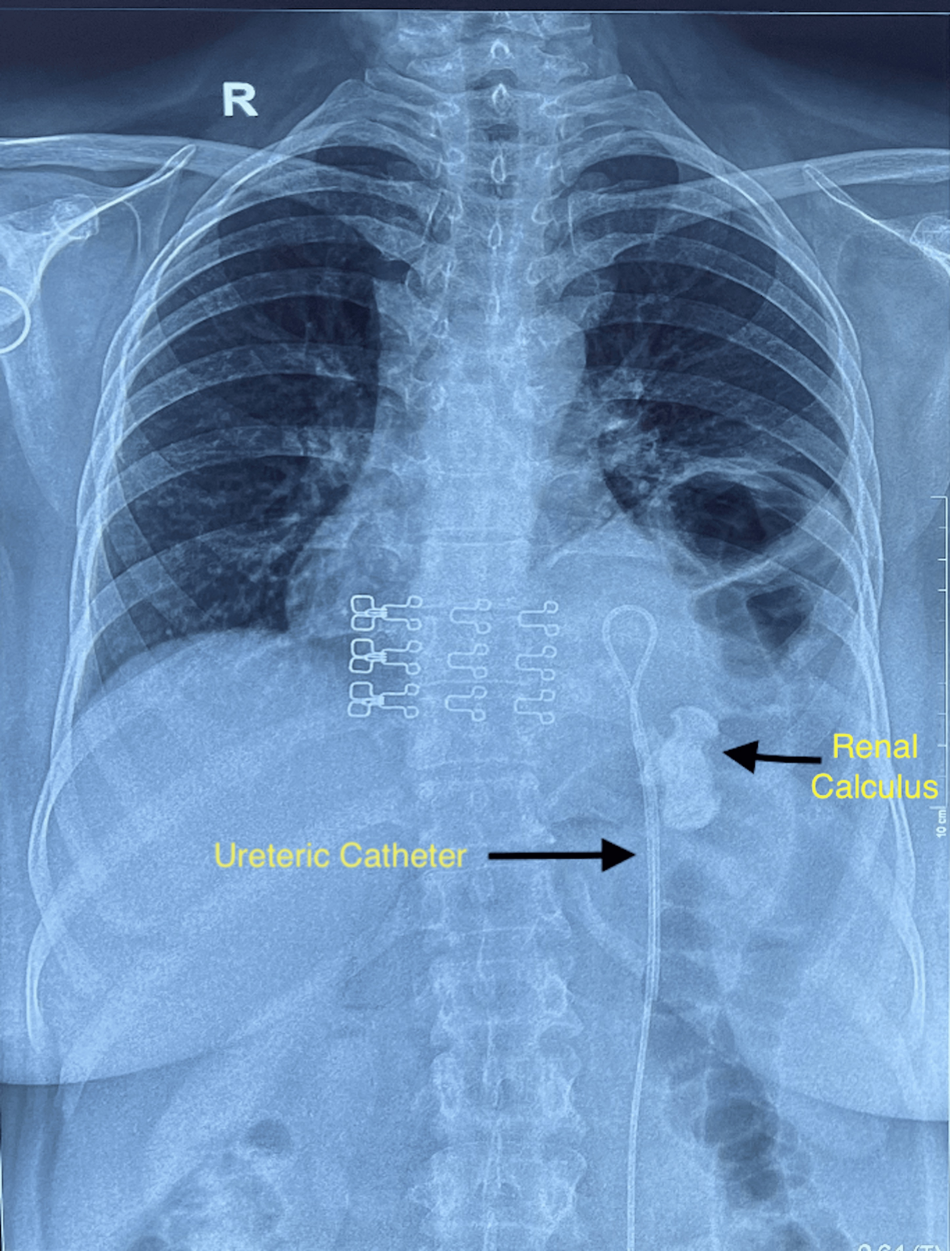
Chest X-ray showing left thoracic kidney with staghorn calculi and ureteric catheter in situ as a stent.

**Figure 2:**
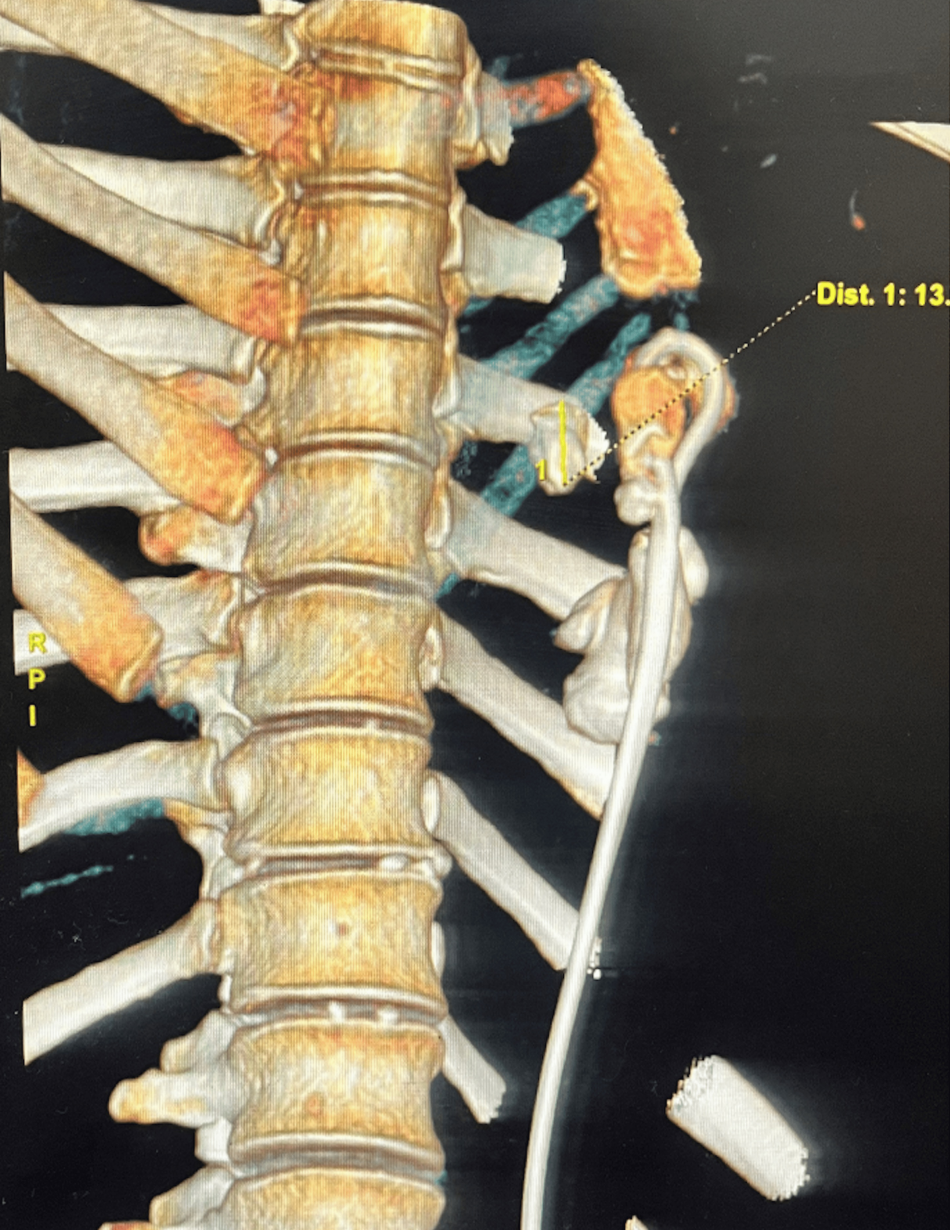
3D reconstruction image (lateral view).

**Figure 3:**
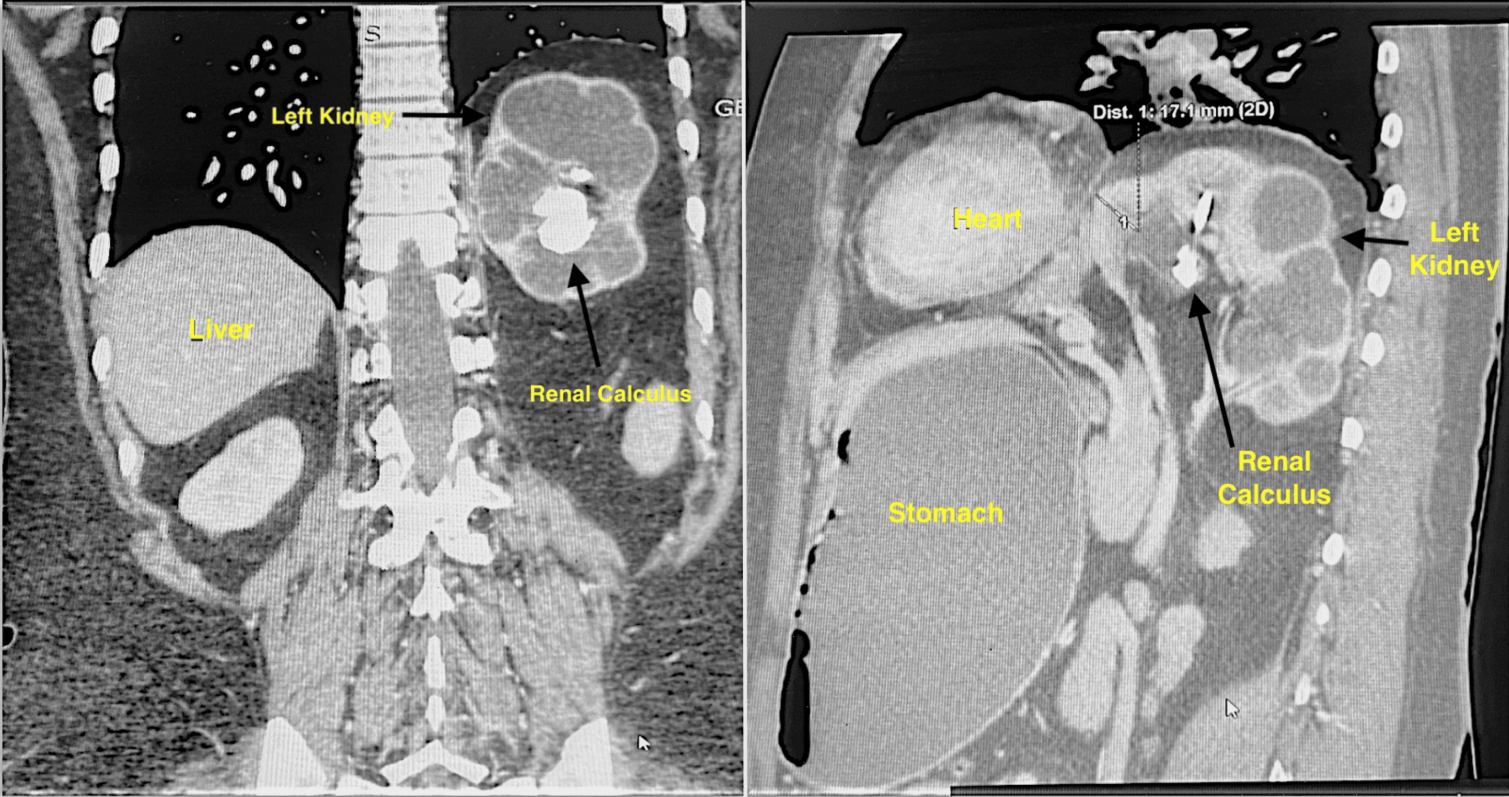
Computed tomography; coronal and sagittal sections (respectively) showing position of the left kidney relative to other organs of the body.

She had no respiratory complaints or previous abdominal surgery. Chest X-ray with kidney-ureter-bladder (KUB) (Figure 1) and contrast-enhanced computed tomography (CECT) chest and abdomen with urography (Figures 2, 3) confirmed a left intrathoracic kidney herniating through a large congenital diaphragmatic defect along with spleen, tail of pancreas, and perinephric fat. A 4 cm staghorn calculus with multiple small calculi was identified.

**Figure 4:**
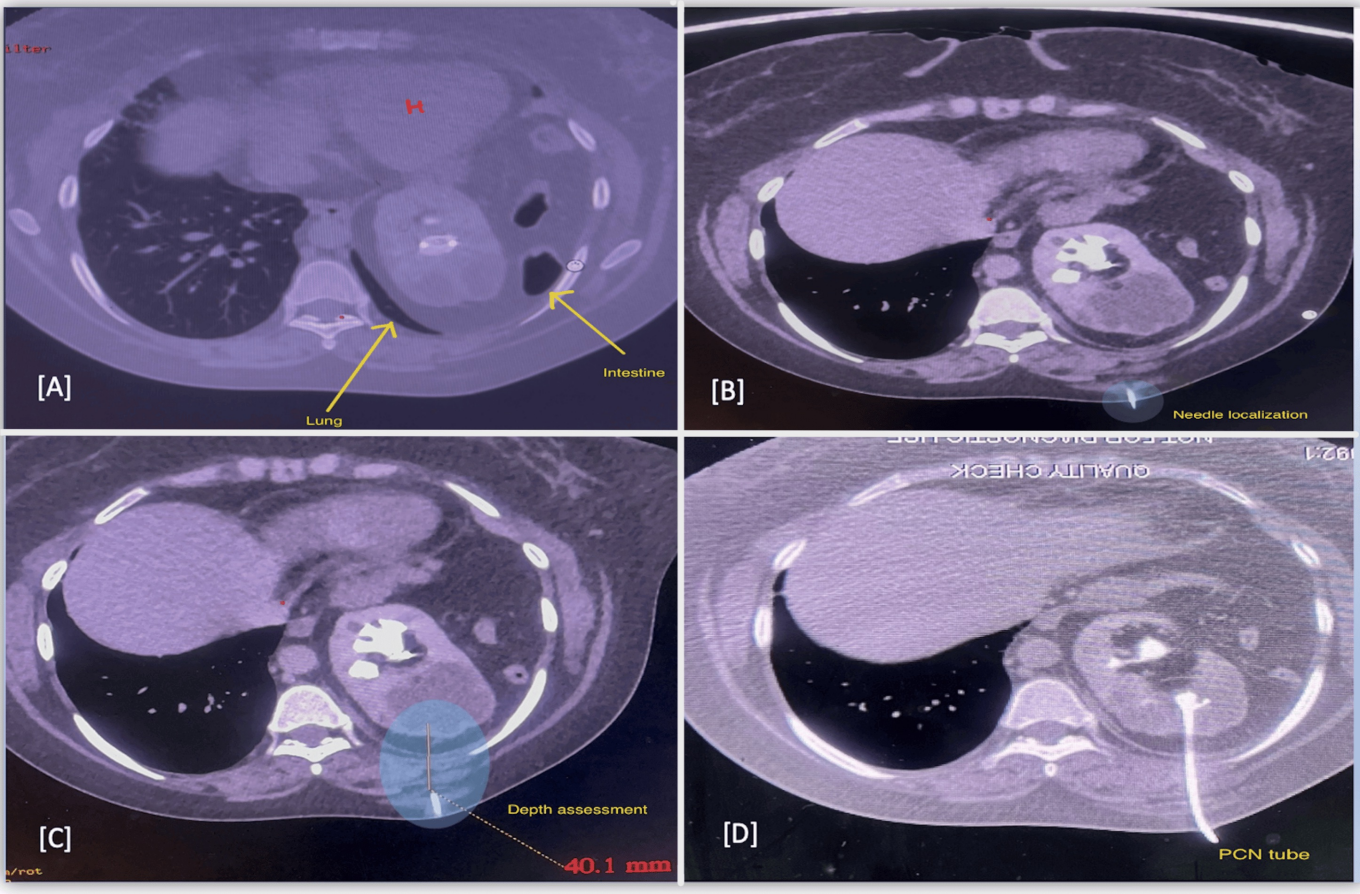
Computed tomography guided left percutaneous nephrostomy placement (axial sections). (A) Narrow window for puncture due to lung on one side and intestine on the other (H: heart); (B) needle localization; (C) depth perception; (D) nephrostomy tube in situ. PCN tube: percutaneous nephrostomy tube.

Renal function tests showed blood urea of 33 mg/dL and serum creatinine of 1.35 mg/dL, which were within normal limits, and urine culture was sterile. Given the presence of calculi in the thoracic kidney, the patient was planned for standard percutaneous nephrolithotomy. In coordination with the interventional radiology department, percutaneous access was established, and a 12 Fr nephrostomy tube was placed into the left posterior superior calyx via the 9th intercostal space under combined computed tomography and ultrasound guidance (Figure 4).

**Figure 5:**
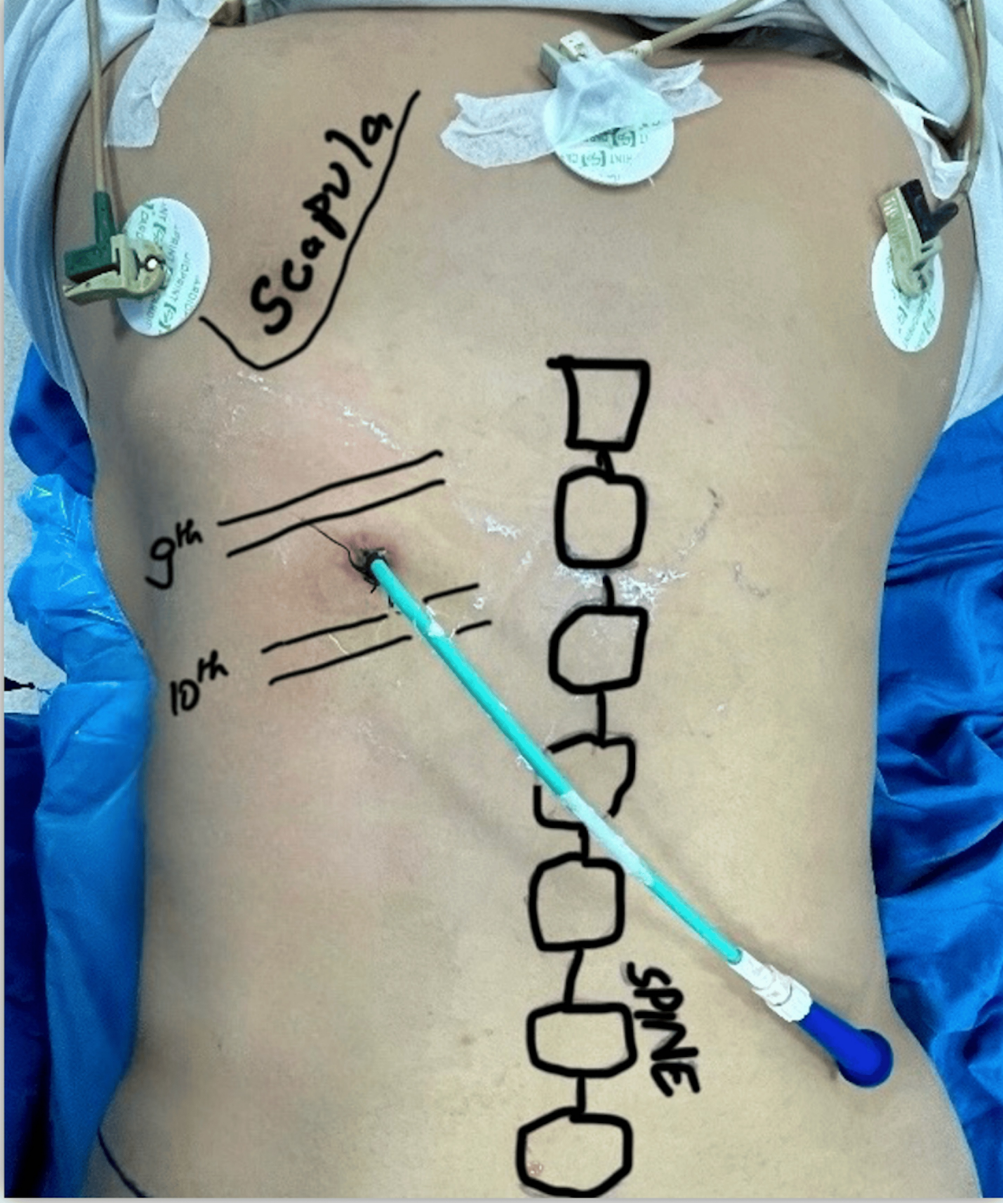
Nephrostomy tube positioned in 9th intercostal space.

**Figure 6:**
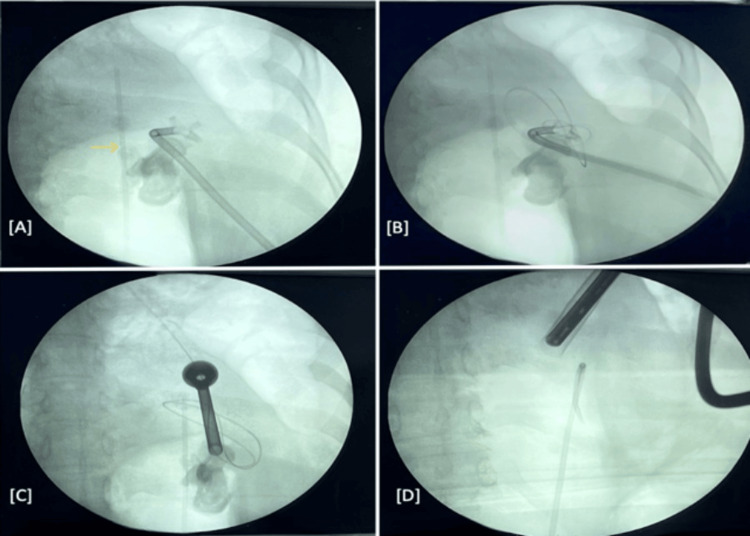
Fluoroscopic view. (A) Left nephrostomy tube in situ with renal calculi and ureteric catheter (yellow arrow); (B) guide wire passed via nephrostomy tube; (C) tract dilated up to 24 Fr; (D) complete clearance.

Prone standard percutaneous nephrolithotomy (PCNL) was performed, and the entry for PCNL was achieved via an already placed nephrostomy tube through the 9th intercostal space (Figure 5). A guidewire was parked via the nephrostomy tube to secure the access, and the nephrostomy tube was removed. Over the guidewire, serial dilatation of the tract up to 24 Fr was done. Stone fragmentation was performed using a pneumatic lithotripter, and complete clearance was confirmed by fluoroscopy and nephroscopy. A 6 Fr ureteric catheter and a 20 Fr nephrostomy tube were placed (Figure 6).

**Figure 7:**
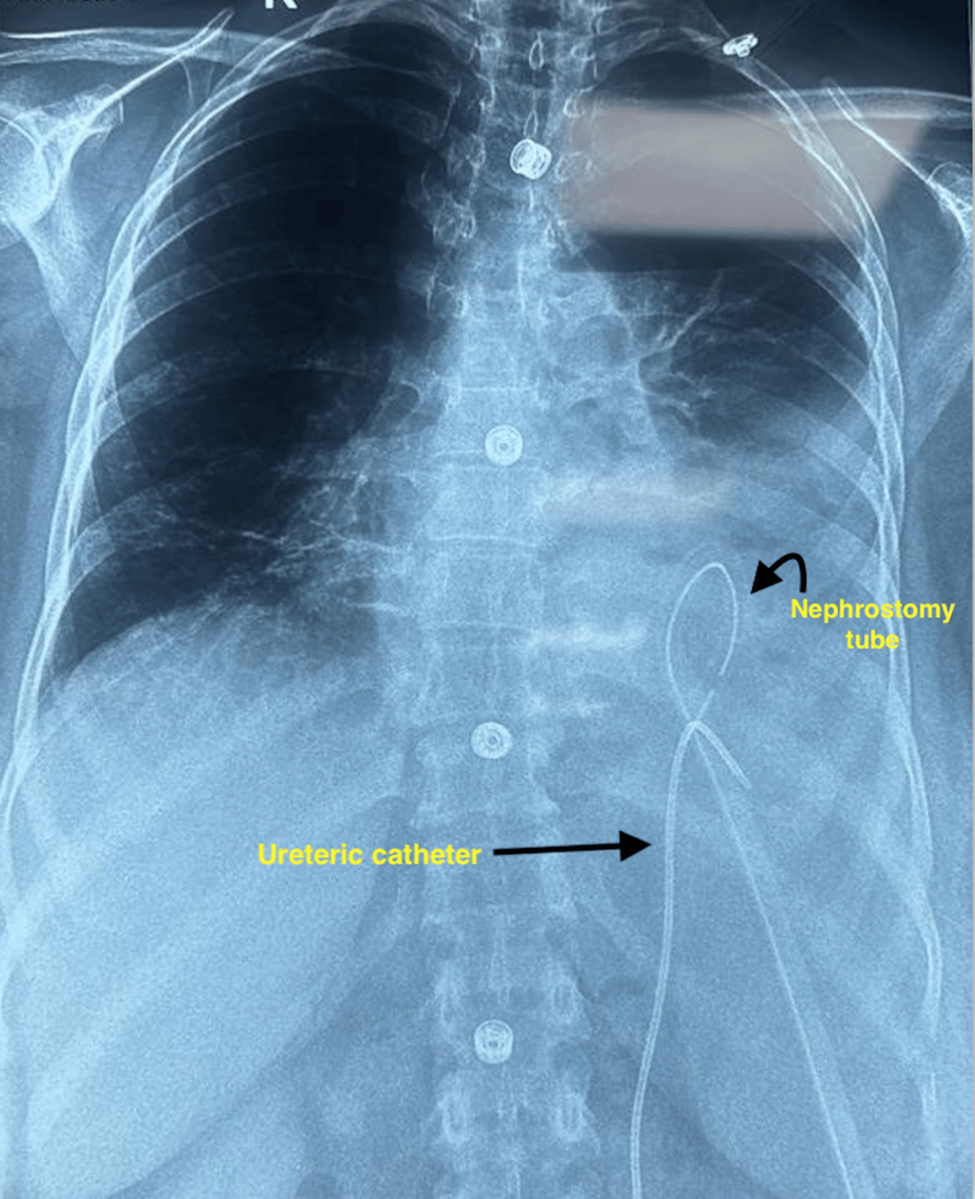
Postoperative X-ray (posterior-anterior view) showing complete clearance of stone burden.

The entire burden of stone was removed via this single tract. Given the unusually long ureter, a ureteral catheter was modified by creating multiple side holes at intervals, and it was placed to function as a stent. The postoperative course was uneventful with no respiratory or surgical complications. A postoperative X-ray (Figure 7) showed no residual calculus.

The nephrostomy tube was removed on postoperative day four, and the patient was discharged on day six.

## Discussion

Ectopic kidney refers to a kidney located outside its normal retroperitoneal position. Its incidence is approximately one in 12,000 clinical cases and one in 900 autopsies [1]. The thoracic kidney is the rarest form, accounting for less than 5% of renal ectopia, with about 200 cases reported worldwide [2].

Embryologically, the kidney normally ascends to the lumbar region by the eighth week of gestation. Incomplete diaphragmatic closure or early renal ascent may result in thoracic renal ectopia [3]. Four types of congenital thoracic renal ectopia have been described: (i) ectopia with intact diaphragm, (ii) diaphragmatic eventration, (iii) diaphragmatic hernia, and (iv) traumatic diaphragmatic rupture [3]. Congenital diaphragmatic hernia associated with an intrathoracic kidney occurs in less than 0.25% of cases, more commonly on the left side [2].

Most thoracic kidneys are asymptomatic and discovered incidentally during imaging for unrelated conditions [4]. The renal vessels often arise more proximally, the ureter tends to be elongated, and the renal hilum faces posteriorly [5]. Despite these anomalies, renal function is typically preserved.

Nephrolithiasis in an intrathoracic kidney is exceedingly rare. The literature describes only two prior cases of successful PCNL performed in such kidneys [4,5]. The unusual position within the thoracic cavity, proximity to vital organs, and altered renal anatomy pose technical and anesthetic challenges for surgical management.

In our case, preoperative planning and coordination with interventional radiology were crucial. Image-guided PCN placement and careful intercostal access allowed safe tract dilation and stone clearance without thoracic complications. This case reinforces that PCNL is feasible and effective for managing calculi in thoracic kidneys when performed with meticulous planning.

## Conclusions

Intra-thoracic ectopic kidney with staghorn calculus represents an extremely rare and surgically challenging condition. Successful management requires comprehensive preoperative imaging, interdepartmental collaboration, and precise intraoperative technique. Minimally invasive procedures such as PCNL can be safely performed in select patients, achieving complete clearance with minimal morbidity.

This case contributes to the limited literature on managing thoracic renal ectopia complicated by nephrolithiasis and highlights that with proper planning, even complex anatomical variations can be approached effectively through minimally invasive means.
